# Machine learning and deep learning applications in microbiome research

**DOI:** 10.1038/s43705-022-00182-9

**Published:** 2022-10-06

**Authors:** Ricardo Hernández Medina, Svetlana Kutuzova, Knud Nor Nielsen, Joachim Johansen, Lars Hestbjerg Hansen, Mads Nielsen, Simon Rasmussen

**Affiliations:** 1 Novo Nordisk Foundation Center for Protein Research, Faculty of Health and Medical Sciences, University of Copenhagen, DK-2200 Copenhagen N, Denmark; 2 Department of Computer Science, University of Copenhagen, DK-2100 Copenhagen Ø, Denmark; 3 Department of Plant and Environmental Sciences, University of Copenhagen, DK-1871 Frederiksberg, Denmark

**Keywords:** Microbiome, Metagenomics

## Abstract

The many microbial communities around us form interactive and dynamic ecosystems called microbiomes. Though concealed from the naked eye, microbiomes govern and influence macroscopic systems including human health, plant resilience, and biogeochemical cycling. Such feats have attracted interest from the scientific community, which has recently turned to machine learning and deep learning methods to interrogate the microbiome and elucidate the relationships between its composition and function. Here, we provide an overview of how the latest microbiome studies harness the inductive prowess of artificial intelligence methods. We start by highlighting that microbiome data – being compositional, sparse, and high-dimensional – necessitates special treatment. We then introduce traditional and novel methods and discuss their strengths and applications. Finally, we discuss the outlook of machine and deep learning pipelines, focusing on bottlenecks and considerations to address them.

## Introduction

All around us, microbial communities are at work. These communities contribute to biogeochemical cycles [1], augment or buffer environmental shifts [2], and are essential to understand disease and health of humans and other organisms [3–6]. Characteristic microbial communities and their metabolites form a dynamic and interactive micro-ecosystem that we call the microbiome [7]. Insights into the workings and relations in these networks hold promise for sustainable agriculture [4, 8], disease prevention and treatment [9], and anthropogenic impact evaluation [10]. A frontier in microbiome research is microbiome engineering to establish a microbiome that supports a desired outcome, be it better health or a higher crop yield [11]. Nevertheless, successful engineering requires knowledge about what constitutes the functioning of a given microbial community, whether certain species within the microbiome are more important than others, and how and to what degree composition and function can be manipulated.

To untangle the complexity of the microbiome, researchers have turned to artificial intelligence. Owing to their powerful predictive and informative potential, machine learning and deep learning have emerged as key tools to advance microbiome research. In this review, we present an overview of how these novel techniques can be used to study the interplay of the microbiome constituents and its links to phenotype.

## Microbiome data types

Even though only a fraction of microbial species can be described through traditional isolation and cultivation approaches [12], advances in omics and high-throughput sequencing have opened the door to a comprehensive description of the microbiome and the generation of large-scale microbiome datasets [13, 14]. The most commonly used methods to analyze the microbiome are amplicon and metagenomic sequencing. In the amplicon methodology, samples are characterized using the reads of specific taxonomic marker genes like the evolutionarily conserved 16 S rRNA gene [15] or the ITS region [16]. Typically, a predefined identity threshold roughly delineates prokaryotic taxa and creates clusters known as operational taxonomic units (OTUs) [17]. Amplicon sequence variants (ASVs) are a newer analog to OTUs. ASVs are generated by a denoising approach and do without an arbitrary dissimilarity threshold, thus allowing resolution of even rare members of the community [18]. In contrast, shotgun metagenomics comprehensively catalogs the totality of genomes within a sample by non-specific sequencing [19]. Through different algorithms, shotgun metagenomic reads can be aligned to curated databases for functional or taxonomic annotation [14]. Furthermore, shotgun metagenomics enables the recovery of metagenome-assembled genomes (MAGs) from the communities using binning strategies such as MetaBAT2 [20] and VAMB that resolve genomes by contig-clustering [21]. Latest advances have even made it possible to characterize the virome, allowing a more comprehensive characterization of the microbiome using shotgun data [22].

These approaches produce feature tables, in which each cell represents the abundance or presence of a specific taxon or function per sample. Whether taxonomic or functional profiles provide a better discriminatory power in downstream analysis is subject to debate [23–25]. In any case, it is due to acknowledge the particularities and challenges related to this data type. Firstly, feature tables are compositional. Compositional data describes relationships between its components, so its parts are not independent and their sum is arbitrary [26, 27]. In addition, feature tables are usually sparse, having excessive zero counts [28], and are high-dimensional, with a larger number of features per sample. This subjects downstream analysis to the curse of dimensionality. The curse is two-fold: a high number of features inflates the computational cost, while a relatively low number of samples impoverishes generalization to other datasets [29].

Different strategies are used to deal with microbiome data. Since common distance and association measures are invalid for compositional data, statistical methods such as log-ratio transformations [26], staying-in-the-simplex approach [30], and calculating component ratios [31] have been established. Traditional log-ratio transformation methods cannot deal with sparsity, so the data is oftentimes imputed; commonly zeros are replaced with pseudo-counts [32]. On the other hand, feature selection and extraction techniques can help overcome the curse of dimensionality. Feature selection entails selecting an optimal subspace of relevant and non-redundant features [33, 34]. In contrast, feature extraction attempts to reduce the dimensionality of a dataset by building a compressed representation of the input features (see examples in further sections). Altogether, the nature of microbiome data demands pre-processing steps that have profound implications on differential feature analysis; arguably, this is bound to affect the performance of machine learning methods [35, 36].

## Machine learning

Machine learning (ML) is a subset of artificial intelligence (AI) methods, which leverage large datasets to recognize, classify, and predict patterns [37]. In microbiome research, ML has been applied to tackle tasks such as phenotyping (namely, predicting an environmental or host phenotype), microbial feature classification (i.e., determining the abundance, diversity, or distribution of the microbiota), studying the complex physical and chemical interactions between the microbiome’s components, and monitoring for changes in the composition of the microbiome [9, 10]. In Table 1, we enumerate select examples of each of these tasks.

**Table 1:** Examples of common tasks and ML methods used in microbiome research.

Task	Predictive goal	Method	Reference
Phenotyping	Sponge bacterial density	Random forests	Moitinho-Silva et al. [42]
Phenotyping	Crop productivity	Random forests	Chang et al. [43]
Phenotyping	Food allergy	Recurrent neural network (LSTM)	Metwally et al. [56]
Phenotyping	Disease (inflammatory bowel disease)	Random forests, lasso, elastic nets	Wirbel et al. [40]
Phenotyping	Disease (e.g., cirrhosis, type 2 diabetes, inflammatory bowel disease)	Convolutional neural networks	Sharma et al. [53], Reiman et al. [54, 55]
Microbial feature classification	Microbiome composition	Autoencoder	García-Jiménez et al. [93]
Microbial feature classification	Metabolic profile	Autoencoder	Le et al. [73]
Interaction analysis	Microbe-metabolite interactions	Embedding	Morton et al. [65]
Interaction analysis	Microbe co-ocurrence patterns	Embedding	Tataru and David [66]
Monitoring composition	Response to diet change	Autoencoder	Reiman and Dai [61]

### Classical methods

Among the classical ML methods, linear regression models, random forests (RFs), and support vector machines (SVMs) have been found to perform well on microbiome data [38, 39]. However, the latter has fallen into disuse in recent studies, relegated to benchmarking. Linear regression methods like lasso and elastic nets model an output, such as a phenotype, as a linear combination of inputs making the interpretation of these methods straightforward. These methods have been recently used in host dysbiosis prediction studies, with comparable results to other methods such as RF [40]. RFs aggregate decision trees, flowchart-like structures constructed by making decisions on how to split a dataset into similar groups. By growing multiple trees from randomly-sampled feature subsets, one can assemble an RF, which has an improved performance over a singular tree [41]. Using microbiome census data, RFs have resolved the symbiont density of sponges [42], predicted maize productivity [43], and differentiated between individuals with or without a substance use disorder [44].

### Dimensionality reduction techniques

Unsupervised ordination methods reduce dimensionality and simplify data for human interpretation. These algorithms are apt for creating visualizations or so-called projections. By computing a linear or non-linear combination of the existing features, these methods generate a compressed representation of the input data. Linear methods, like principal component analysis (PCA) and principal coordinate analysis (PCoA), are popular tools to visualize and contrast microbial communities, such as identifying the habitat or geographic origin of microbiota samples [45, 46]. Methods like t-stochastic neighbor embedding (t-SNE) and uniform manifold approximation and projection (UMAP) faithfully capture and reveal local and non-linear relationships in complex microbiome datasets, but their tuning is finicky [47–49].

## Deep learning

Deep learning (DL) is a class of ML algorithms that involves various artificial neural network architectures. DL models rely on nodes (also called neurons or units), which are functions that transform inputs and forward the outputs to other nodes. The connections between nodes result in a network consisting of multiple layers (hence the name deep neural networks), which can be connected and organized in different layouts, or architectures.

The most basic neural network architecture is the fully-connected neural network (FCNN), in which the nodes from one layer are fully connected to every node from the subsequent layer. Lo and Marculescu [50] employed this architecture to predict host phenotype from raw metagenomic count data, obtaining better classification accuracy over traditional methods across different datasets. While the FCNN is an effective standalone model, it is most often the basic building block of more complex architectures.

### Picturing microbiomes

**Fig. 1:**
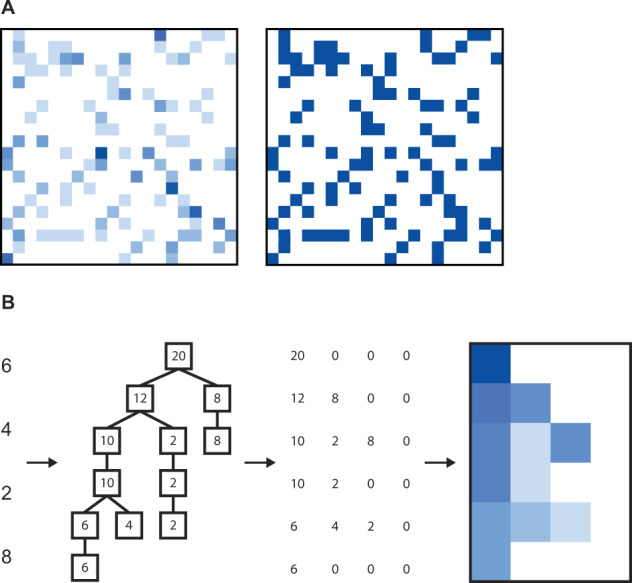
Examples of CNN image inputs generated from OTU tables. **A** The image is filled with species abundances (left) or presences (right). **B** For a single sample, the phylogenetic tree is constructed, populated with species abundances, and rearranged into a matrix.

Researchers have found creative ways to enrich OTU abundance matrices with spatial information (such as that inherent in phylogenetic trees). By doing so, they can leverage the inductive capabilities of convolutional neural networks (CNNs). CNNs excel at summarizing local structure in their input; thus, they are well-suited to handle data conveying spatial information, such as images. Nguyen et al. [51, 52] rendered an OTU table into an image by reshaping each sample into a square, where each pixel was colored based on the abundance or presence of microbial taxa (Fig. 1A). taxoNN rearranges an OTU table based on its inherent phylogenetic information [53], whereas PopPhy-CNN [54, 55] populates a phylogenetic tree with OTU abundances, and then transforms the tree into a two-dimensional matrix (Fig. 1B). Generally, these approaches have outperformed their benchmarks (both traditional ML methods and FCNNs) in the task of host phenotype prediction.

### Examining patterns in temporal data

Recurrent neural networks are mostly used to explore sequential or historical patterns. These architectures are oftentimes chain-like, consisting of loops that pass the information from one point in time to the next. In microbiome studies, RNNs allow the prediction of temporal dependencies and dynamic patterns. Metwally et al. [56] were one of the first to build a predictive model based on longitudinal microbiome profiles. Based on data from a study tracking infants’ allergic phenotype over three years, their model was built to predict food allergy, outperforming traditional ML models and FCNNs, but not reaching a performance suitable for clinical utilization. phyLoLSTM [57], an RNN-based framework, improves on previous classification accuracy by using taxoNN for feature extraction. Around the same time, Chen et al. [58] proposed a different time-aware framework, combining imputation of inconsistent temporal data and feature engineering to enrich the input tables with phylogenetic information. Their method was tested on multiple longitudinal microbiome datasets, with the task to predict different host statuses: such as type of diet, nationality, food allergy, disease, and drug use.

### Unveiling latent information

For the sake of computational cost and efficiency, it is often beneficial to reduce the dimensions of microbiome feature tables. In DL, this low-dimensional latent representation is called an embedding, and it is often created with an autoencoder [59]. The autoencoder architecture consists of an encoder network that learns a latent representation of the supplied input and a decoder network that tries to reconstruct the input from this representation. By minimizing the difference between original and reconstructed data, the network learns to faithfully compress information. DeepMicro [60] presents multiple autoencoder variations and how each different latent representation improves prediction of irritable bowel syndrome and type 2 diabetes.

The modularity of autoencoders enables multimodal-data integration, holding promise for better and more comprehensive models. As presented by Reiman and Dai [61], a bimodal autoencoder can integrate diet and microbial composition to predict the microbial dynamics response to dietary change. Grazioli et al. [62] introduce a disease prediction model that relies on the product-of-experts approach to integrate the information from two autoencoders, each expert on a different modality: abundance (species-level) and presence (strain-level) features, respectively.

Other algorithms that produce embeddings draw inspiration from word processing methods, such as word2vec [63] and GloVe [64]. These methods can create dense embeddings that capture co-occurrence patterns [65, 66]. Such representations summarize the relations in the microbiome samples (e.g., microbe-metabolite interactions) and are useful for host-phenotype classification tasks.

## Outlook

### Bottlenecks for further applications

Even though ML was promised as a powerful predictive tool in microbiome research, it is challenged by various obstacles that limit its wide and readily application [67]. Common limitations have to do with interpretability, data hungriness, and model evaluation and selection. Plainly, ML empirically establishes a link between an input and a response without any mechanistic understanding of the underlying logic behind such a relationship. This has led to ML models being generally regarded as black boxes with inexplicable innards. The issue becomes evident, for instance, in clinical decision-making, where mechanistic insight is instrumental to trust causal inference [67]. Although the concept of interpretability is ill-defined, there is growing interest in interpretable ML [68]. For instance, the deep forest algorithm ranks features by importance and has already been explored in microbiome-wide association studies [69, 70]. Zhu et al. also proposed an approach to embed a microbial interaction network into an FCNN, thus constraining the learning process with *a priori* knowledge [71]. Other frameworks, such as DeepCoDA [72], prioritize feature attribution by relying on linear transformations, whereas SparseNED, an encoder-decoder model, has been used to capture microbe-metabolite relationships associated with inflammatory bowel disease through a sparse and interpretable latent space [73]. More generally-applicable ways to open the black box are thoroughly reviewed by Guidotti et al. [74].

The second hurdle is the dearth of voluminous, high-quality, and correctly-labeled data required to reliably train ML models [75–78]. Adadi [78] highlights strategies to tackle the issue of data availability of ML, including data augmentation, non-supervised learning, transfer learning, and hybrid models. Data augmentation comprises a set of practices to create synthetic samples. Lo and Marculescu [50] modeled and sampled microbiome profiles from a negative binomial distribution to enlarge their training dataset and improve the host phenotype classification performance of their FCNN model. Sayyari et al. [79] addressed the pervasive limitation of low-sample numbers and under-represented classes by introducing a tree-based associative data augmentation (TADA) approach to generate new OTU samples from an inferred phylogenetic tree. The non-supervised learning paradigm encompasses semi- and unsupervised learning approaches (think autoencoders), which are less reliant on labeled samples. Transfer learning and hybrid learners are yet to be explored in the context of microbiome research.

A paramount consideration is data quality, and, as such, our advice is to be aware of the source, deficiencies, and biases of the microbiome dataset [80]. Techniques to curb this obstacle include deduplication, class balancing, outlier removal, and imputation. These techniques influence a model’s performance, as noted by Chen et al. [58], who assay the effect of different imputation techniques on longitudinal microbiome data. Even though the collection of large and properly-annotated sample sizes is difficult to overcome in the microbiome setting, researchers can (after ensuring samples are collected and processed under the same regime) aggregate data from multiple studies, allowing the study of cohort-dependent effects [40, 81]. In any case, we stress that ML models are tightly dependent on their training dataset, so special attention should be paid to the data that feeds them.

An additional challenge microbial ecologists face has to do with the evaluation, selection, and tuning of the appropriate ML model for a given task. While choosing among the many models and fishing for a set of suitable hyperparameters seems like a daunting task, we encourage aspiring ML partakers to take advantage of the fertile ML ecosystem. Implementation has been facilitated by continuous development of Python and R libraries, such as scikit [82], PyTorch [83], Tensorflow [84], and mlr3 [85]. Moreover, high-level frameworks, like FastAI [86], PyTorch Lightning [87], and Keras [88], make implementation even more approachable. Tuning and developing ML models should also take advantage of existing frameworks for generating synthetic microbiome datasets like those provided by the CAMI consortium [89]. Not only will synthetic and pre-labeled microbiomes help guide the choice of hyperparameters and model design, but it also provides a basis for benchmarking and comparison. Comparison across multiple datasets enables assessing the robustness of ML methods, but, as remarked in neutral benchmarking studies [90], the selection of a reference dataset is critical to ensure fair comparisons.

**Fig. 2:**
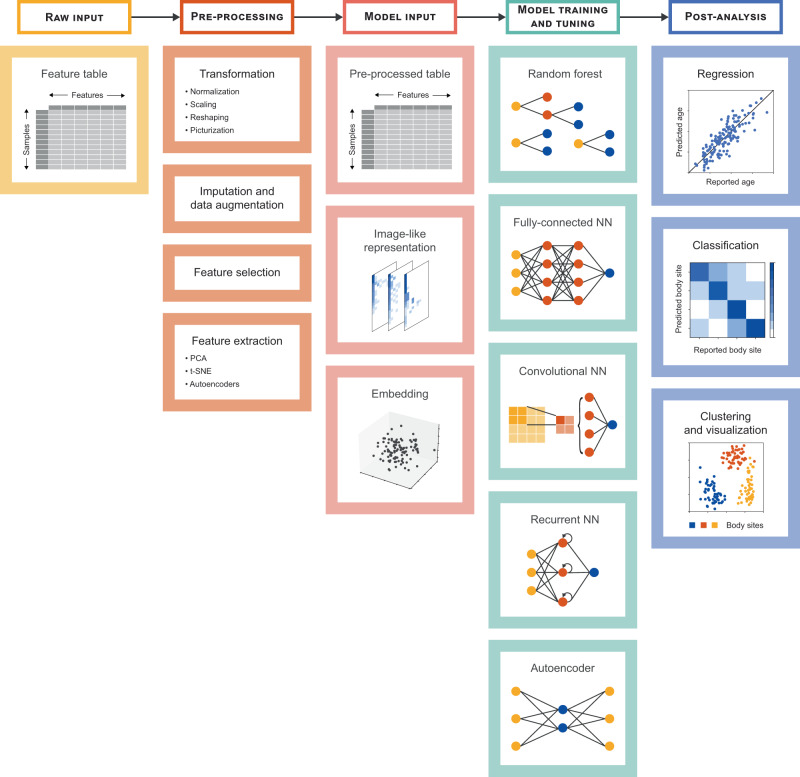
Key steps of ML-assisted microbiome analysis. Generally, analysis begins with a feature table describing the functional or taxonomic profile of a microbiome. As part of the pre-processing step, this table can be transformed, imputed, or augmented, among other processes. The outcome of pre-processing can be tabular data or a set of image-like representations or embeddings per sample. The next step entails training and tuning ML or DL models, such as random forests, fully-connected neural networks, convolutional neural networks, recurrent neural networks, and autoencoders. Finally, the results help to elucidate the link between the microbiome composition and a continuously- (regression) or discretely-described (classification, clustering, and visualization) phenotype.

Lastly, we summarize the key steps of ML-assisted microbiome analysis in Fig. 2, and provide the following quick tips and heuristics:

1. Get familiar with the dataset. An early inspection of the input data can help gauge the size of the feature space, identify whether the dataset contains unbalanced classes, or determine if imputation or feature engineering is an option.
2. Set up a model selection and benchmarking strategy. Either split the dataset into training, validation, and test subsets (in the case of a large dataset) or plan for cross-validation (for smaller datasets). Select appropriate metrics to compare models and estimate their performance.
3. Choose the appropriate method. Although the choice is data- and task-dependent, traditional ML algorithms are good starting points, as they require minimum tuning and are relatively easy to implement. If large-scale or multi-modal data is available, consider a DL approach like an autoencoder to incorporate all data facets into informative embeddings. In the case of sequential data with a longitudinally-profiled microbiome, try an RNN framework that is suitable for capturing temporal dependencies. If spatial information can be embedded into the input such as a phylogenetic tree that can be decomposed into a 2D matrix, consider CNNs.

### Novel techniques to keep on the watchlist

A comprehensive evaluation of DL models by LaPierre et al. suggests that it is likely that the upper limits on predictive accuracy from only metagenomic data have been reached [91]. Nonetheless, previous research has demonstrated improved predictive power can be attained by marrying different data modalities, such as microbiome, genetic, and environmental data [92]. For instance, García-Jiménez et al. [93] implemented a concept of multimodal embedding by minimizing the distance between the two latent spaces created by the separate encoders of two modalities (environmental variables and microbial composition). A lineage of work on multimodal variational autoencoders investigates the most suitable way of combining the latent spaces of individual modalities depending on the dataset properties [94–99]. Although multimodal VAEs [96] have been used to analyze single-cell multi-omics data [100], to the best of our knowledge, this kind of learner has not yet been applied to multi-omics microbiome data.

## Conclusions

The study of microbial communities is lush. Amplicon and metagenomic sequencing produce feature tables that taxonomically or functionally describe a microbiome, and that, with appropriate labels, can fuel ML and DL-based methods. DL models are powerful tools with a wide array of applications in the field of microbiome research. Notably, these methods enable linking specific taxa to a host phenotype or monitoring the dynamics and host response to changes in the composition of the microbiome. Although different configurations of ML and DL models exist, the choice is task and input-dependent. In this review, we have not only provided examples of applications of AI in the realm of microbiome research but also presented a list of considerations to heed when using these models. Further research into the current bottlenecks of data availability and model interpretability will further propel the use of DL in microbiome studies and expand our understanding of the microbial interactions that shape our world.

## Data Availability

Data sharing not applicable to this article as no datasets were generated or analysed during the current study.

## Glossary

Architecture: Organization and size of the layers of a neural network.
Autoencoder: A neural network consisting of an encoder-decoder pair. The encoder reduces the dimensionality of the input, thus creating a so-called latent representation; whereas, the decoder is tasked with generating a reconstruction of the original input from such latent space.
Benchmarking: The practice of comparing the performance of different approaches using a reference dataset.
Compositional data: Data with the following characteristics: the total sum of each component is defined by the sampling technique (such as the capacity of a high-throughput sequencing instrument), and the difference between values is only meaningful proportionally (that is, values are relative to an arbitrary total and not independent).
Convolution: A mathematical operation between an input matrix and filter matrix of the same rank, consisting of multiplication between a slice of the input and a filter and subsequent summation of the resulting product.
Convolutional layer: A layer that uses the convolution operation. They are widely used to capture spatial relationships within a sample’s features and are suited for image classification.
Elastic net: A linear regression method with regularization that shrinks both irrelevant and outlier coefficients towards zero. Such shrinkage prevents overfitting, making the model more generalizable to other datasets.
Embedding: Typically, a low-dimensional continuous-valued representation of the feature space.
Feature: An input variable of a model.
Fully-connected layer: An abstraction of a matrix multiplication between an input matrix and a weight matrix, representing a connection between every input and output node.
Label: A categorical or binary output of a model.
Lasso: A linear regression method with regularization to shrink irrelevant coefficients towards zero. This regularization is useful to handle highly-sparse datasets.
Layer: An abstraction of a numerical transformation.
Machine learning paradigm: A machine learning pattern. There are two main paradigms used in microbiome research: unsupervised learning (learning from unlabelled data) and supervised learning (learning from labeled data). Other paradigms include semi-supervised learning and transfer learning.
Random forest: An aggregated collection of independently-trained decision trees, where each decision tree is trained on a randomly-sampled subset of the training dataset.
Recurrent layer: An extension of the fully connected layer that is looped multiple times. As each run feeds from a previous state, this layer excels at capturing time dependencies present in sequential or longitudinal data.
